# Transcriptome and Proteome Analysis Revealed the Influence of High-Molecular-Weight Glutenin Subunits (HMW-GSs) Deficiency on Expression of Storage Substances and the Potential Regulatory Mechanism of HMW-GSs

**DOI:** 10.3390/foods12020361

**Published:** 2023-01-12

**Authors:** Yun Zhao, Jie Zhao, Mengyun Hu, Lijing Sun, Qian Liu, Yelun Zhang, Qianying Li, Peinan Wang, Wujun Ma, Hui Li, Huimin Gao, Yingjun Zhang

**Affiliations:** 1 Laboratory of Crop Genetics and Breeding of Hebei, Institute of Cereal and Oil Crops, Hebei Academy of Agriculture and Forestry Sciences, Shijiazhuang 050035, China; 2 College of Agronomy, Qingdao Agricultural University, Qingdao 266109, China; 3 Food Futures Institute, College of Science, Health, Engineering and Education, Murdoch University, Perth 6150, Australia; 4 Institute of Cash Crops, Hebei Academy of Agriculture and Forestry Sciences, Shijiazhuang 050051, China

**Keywords:** wheat, HMW-GSs, seed storage substances, regulation mechanism, candidate genes

## Abstract

The processing quality of wheat is affected by seed storage substances, such as protein and starch. High-molecular-weight glutenin subunits (HMW-GSs) are the major components of wheat seed storage proteins (SSPs); they are also key determinators of wheat end-use quality. However, the effects of HMW-GSs absence on the expression of other storage substances and the regulation mechanism of HMW-GSs are still limited. Previously, a wheat transgenic line LH-11 with complete deletions of HMW-GSs was obtained through introducing an exogenous gene *Glu-1E^b^x* to the wild-type cultivar Bobwhite by transgenic approach. In this study, comparative seed transcriptomics and proteomics of transgenic and non-transgenic lines at different seed developmental stages were carried out to explore the changes in genes and proteins and the underlying regulatory mechanism. Results revealed that a number of genes, including genes related to SSPs, carbohydrates metabolism, amino acids metabolism, transcription, translation, and protein process were differentially enriched. Seed storage proteins displayed differential expression patterns between the transgenic and non-transgenic line, a major rise in the expression levels of gliadins were observed at 21 and 28 days post anthesis (DPA) in the transgenic line. Changes in expressions of low-molecular-weight glutenins (LMW-GSs), avenin-like proteins (ALPs), lipid transfer proteins (LTPs), and protease inhibitors (PIs) were also observed. In addition, genes related to carbohydrate metabolism were differentially expressed, which probably leads to a difference in starch component and deposition. A list of gene categories participating in the accumulation of SSPs was proposed according to the transcriptome and proteome data. Six genes from the MYB and eight genes from the NAC transcription families are likely important regulators of HMW-GSs accumulation. This study will provide data support for understanding the regulatory network of wheat storage substances. The screened candidate genes can lay a foundation for further research on the regulation mechanism of HMW-GSs.

## 1. Introduction

Cereal production is the foundation of human food security. Cereals comprise 70–75% carbohydrates and 6–15% protein, accounting for 50% of human energy intake [1]. Wheat is one of the most important cereals. In 2021, the world production of wheat reached 770 million tons (FAOSTAT, https://www.fao.org/faostat/ (accessed on 1 January, 2023)). Of total wheat grain proteins, around 80% are prolamins, consisting mainly of glutenins and gliadins that determine the use and end-product quality [2,3]. The high- and low-molecular-weight glutenin subunits (HMW-GSs and LMW-GSs) form glutenin macropolymers (GMPs) to confer dough viscoelasticity [4]. The composition of different HMW-GSs greatly influences the rheological quality of wheat dough. For example, glutenin subunits 7* + 8 at the *Glu-B1* locus and 5 + 10 at the *Glu-D1* locus can produce strong wheat dough [5,6]. Gliadins are reported to increase dough extensibility while decreasing strength, and different gliadin fractions contribute differently to this defect effect [7].

In addition to prolamins, a number of other proteins are also identified in wheat grains, including farinins, purinins, triticins, globulins, grain softness-related proteins (GSPs), amylase/protease inhibitors, serpins, beta-amylases, tritins, and numerous enzymes [8,9]. Those non-gluten proteins mainly participate in grain development, maturation, desiccation, and plant defense processes. Furthermore, they also directly or indirectly affect wheat processing quality. The bakery industry uses α-amylase to reduce dough elasticity and attenuate the negative effects of highly damaged starch on dough properties [10]. Avenin-like b proteins can potentially increase sodium dodecyl sulfate sedimentation (SDSS) and dough elasticity [11]. Protease inhibitors positively contribute to SDSS and crude protein content [12]. Grain softness proteins are major determinants of grain hardness [13]. Despite the positive effects of glutenins and other SSP constituents on processing quality, a large number of wheat SSPs are immunogenic and pose negative effects on human health [14]. This immunogenic toxicity has been characterized in gliadins, glutenins, amylase/trypsin inhibitors (ATIs), and non-specific lipid transfer proteins (nsLTPs) [15]. To reduce the amounts of the immunogenic proteins, attempts have been made to silence some of the gliadin constituents [16,17,18].

Wheat prolamins belong to a large multigene family, it has been estimated that bread wheat contains over 200 prolamin genes, including six HMW-GS genes, ~80 LMW-GS genes, and ~150 gliadin genes [19,20,21]. Moreover, there are considerable allelic variations among those prolamin genes. These increase the difficulty of clarifying the prolamins regulatory network. The expression of SSPs is mainly regulated on the transcriptional level. A series of different transcriptional regulation mechanisms have been proposed and verified in recent years. Different types and copy numbers of cis-elements can determine the expression status and quantity of different SSPs. The conserved cis-elements in the promoters of *Glu-3* [22] and *Glu-1* [23,24] are well investigated and characterized, suggesting various conserved cis-elements exist in SSP genes. A number of significant transcriptional factors (TFs) have been identified that directly or indirectly interact with these cis-elements. The main TFs involved belong to DOF [25], bZIP [26], MYB [27,28], and NAC [29] families. They either act independently or interact with each other [27,30] to initiate the gene expressions of SSPs. Due to the more positive contribution to dough quality and less complexity, a higher number of regulation-related genes for HMW-GSs than for gliadins and LWM-GSs have been identified. Multiple homoeologs/paralogs and high sequence similarity in gliadins and LWM-GSs hinder the research progress in their expression regulation [14]. In transcriptional regulation, the expression profiles of targeted genes and potential TFs are always correlated. Coexpression analysis between TFs and SSPs in *Triticum urartu* revealed a total of 71 TFs that belong to 23 families co-regulated with all SSPs [29]. Therefore, understanding the complex regulation mechanisms will facilitate the optimization of SSP constituents in wheat quality breeding.

Previously, a transgenic line LH-11 with deficiency of HMW-GS genes was obtained by transferring an exogenous *Glu-1E^b^x* gene from *Thinopyrum bessarabicum* to wheat variety Bobwhite. The total gliadin content increased in LH-11 compared to the wild-type. Deletion of HMW-GSs also significantly changed the content of different LMW-GSs and gliadin fractions. The wet gluten content, sedimentation value, dough development time, and stability time of LH-11 were remarkably lower than that of Bobwhite [31]. In the current study, comparative seed transcriptome and proteome profilings of the transgenic and non-transgenic lines were carried out to explore (1) what happened inside the LH-11 seed, (2) the effects of silence of HMW-GSs on the expression of other storage substances, and (3) the regulation mechanism of HMW-GSs. The results will provide data support for revealing the complex regulatory network of wheat storage substances.

## 2. Materials and Methods

### 2.1. Plant Materials

The transgenic line LH-11 [31] that was generated by transforming an exogenous *Glu-1E^b^x* gene (GenBank accession AY525782) into a common wheat cultivar Bobwhite through particle bombardment was used in the current study. The introduction of *Glu-1E^b^x* gene which encodes the HMW-GS of *Thinopyrum bessarabicum* resulted in a co-suppression of both the *Glu-1E^b^x* and the wheat endogenous HMW-GS encoding genes. Seed samples were collected from both Bobwhite and LH-11 at developmental stages of 7, 14, 21, and 28 DPA. Plants were planted in the field station of the Institute of Cereal and Oil Crops, Hebei Academy of Agriculture and Forestry Sciences, Shijiazhuang, China in 2021.

### 2.2. Transcriptome Analysis

BGISEQ-500 platform (Beijing Genomics Institute, Shenzhen, China) was used for transcriptomics analyses. The software SOAPnuke was used to filter the raw data and obtain the clean reads. Adapter reads, reads with over 5% unknown bases, and low-quality reads which had over 20% reads with quality score lower than 15 were removed to obtain clean reads. HISAT2 (version 2.1.0) [32] and Bowtie2 (version 2.2.5) [33] were used to align the clean reads to the reference sequence, and RSEM (version 1.2.8) [34] was used to calculate the expression levels of genes and transcripts. Differentially expressed genes (DEGs) were classified using the official annotation and classification. The phyper function in R was used for Gene Ontology (GO) and Kyoto Encyclopedia of Genes and Genomes (KEGG) pathway enrichment analysis. The gene expression level of RNA-Seq was estimated by the reads per kilobase per million mapped reads (RPKM). Software DESeq2 (version 1.26) [35] was employed to analyze the differential gene expression, and genes with *p* < 0.05 were identified as DEGs.

### 2.3. DIA Proteome Analysis

Mass spectrometry data were obtained using the DIA (data independent acquisition) mode. Wheat grain samples from different developmental stages were used for protein extraction using similar procedure as described in Lv’s study [36]. All the proteins were hydrolyzed by trypsin (enzyme/protein = 1:40 *w*/*w*), then the peptides were separated with UltiMate 3000 UHPLC (Thermo Fisher Scientific, Waltham, MA, USA). Data-dependent acquisition (DDA) on a mass spectrometer Q-Exactive HF (Thermo Fisher Scientific, USA) was used to generate the spectral library followed by the individual sample detection with DIA mode. The software MaxQuant [37] and MSstats [38] were used for the identification and quantification of peptides and proteins with the UniProt Swiss-Prot protein database, NCBI and Ensemble gene annotation databases. Differentially expressed proteins (DEPs) at fold change ≥ 2 and *p* value < 0.05 were identified and enriched. The GO and KEGG enrichments were performed as described in the transcriptome analysis.

### 2.4. Gene Expression Analyses

The total RNA was isolated using the Trizol reagent (www.tiangen.com (accessed on 10 December 2020)) according to the manufacturers’ instructions as described in Sun’s study [39]. About 100 mg of wheat seed sample was fully ground in liquid nitrogen, and 1 mL of TRNzol Universal reagent was added. The homogenate was placed at room temperature for 5 min. Add 0.2 mL chloroform, shake vigorously, then centrifuge at 12,000 rpm at 4 °C for 15 min, transfer the water phase to a new centrifuge tube, add equal volume isopropanol, mix well, and place it at room temperature for 10 min. Centrifuge at 12,000 rpm at 4 °C for 10 min to remove the supernatant. Add 1 mL of 75% ethanol to wash the precipitate. After drying at room temperature, add 100 μL of RNase-Free ddH_2_O to fully dissolve the RNA. The Primer Premier 5.0 (http://www.premierbiosoft.com (accessed on 5 December 2020)) was used to design specific primers. The RT-PCR was carried out in total volumes of 20 µL with SYBR Premix Ex Taq (https://www.takarabiomed.com.cn (accessed on 11 December 2020)) in a CFX96™ real-time PCR detection system (BIO-RAD, Hercules, CA, USA). *Actin* as an internal control gene was used to normalize the mRNA expression level. The *actin* expression was stable and did not change across all of the RNAseq datasets. Three biological replicates of RT-qPCR were performed for each sample. The average values of 2^−ΔCt^ were used to identify differences in gene expression. Primers used in the current study are listed in Supplementary Table S11.

## 3. Results

### 3.1. Few Genes Were Commonly Identified in Transcriptome and Proteome

**Figure 1:**
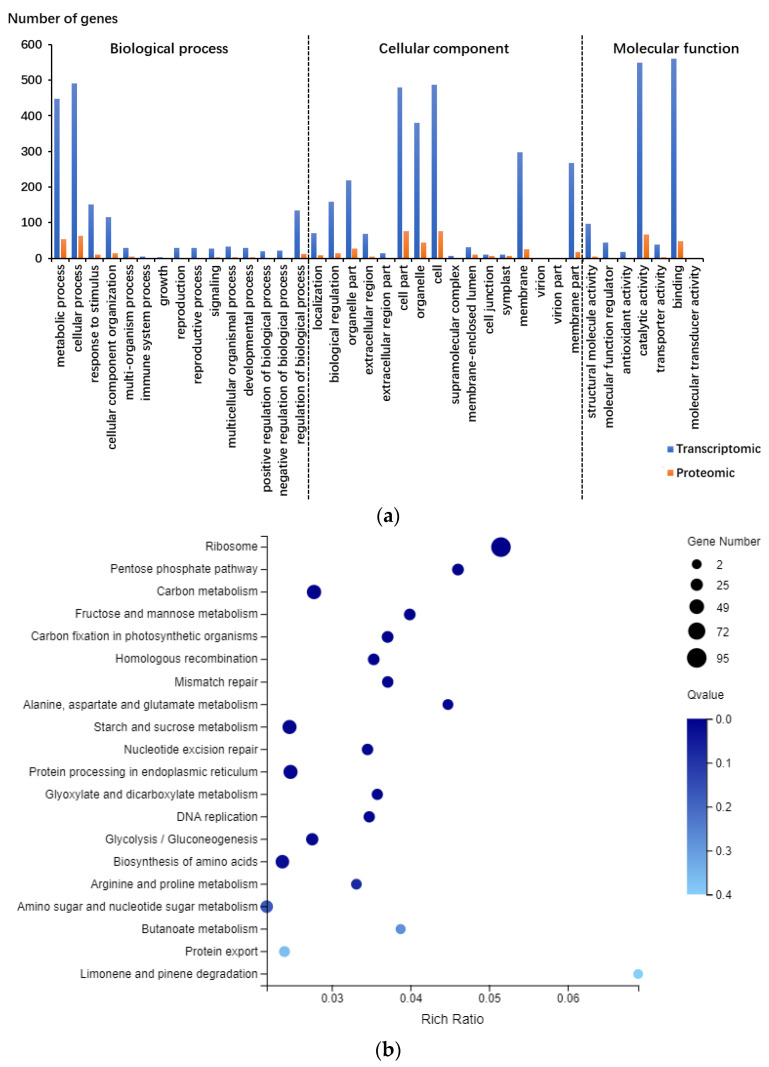

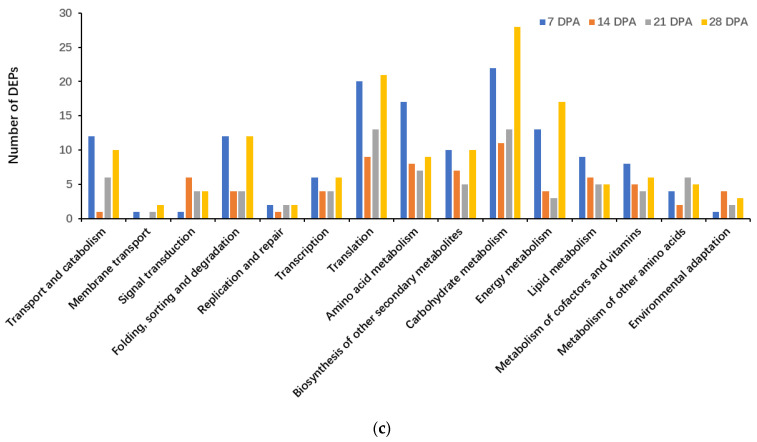
GO and KEGG enrichment results. (**a**) GO enrichment results from both transcriptomic and proteomic data at 21 DPA. (**b**) KEGG enrichment results at 21 DPA from the transcriptomic data. (**c**) KEGG enrichment results at 7, 14, 21, and 28 DPA from the proteomic data.

In total, 2962 genes were differentially expressed. Among the four different stages (Supplementary Table S1), more DEGs were observed at 21 and 28 DPA (1591 and 1344) compared with 7 and 14 DPA (237 and 198) (Table 1 and Supplementary Figure S1). A total of 36 DEGs were repeatedly identified at all four stages (Supplementary Table S3). Among them, five genes are involved in the RNA progenesis/surveillance process, they are TraesCS4A02G036900, TraesCS5A02G434400, TraesCS7A02G153300, TraesCSU02G117600, and TraesCSU02G119700, all containing the RNA/DNA recognition motif. The GO analysis of DEGs suggested that, genes with binding and catalytic activity that participated in cellular and metabolic processes were enriched at all four stages (Figure 1 and Supplementary Figure S2). The KEGG analysis of DEGs suggested that at 7 DPA, the enriched genes are involved in lipid metabolism, carbohydrate biosynthesis process, mitotic cell cycle process, DNA integration, and carboxylyase activity. At 14 DPA, the enriched genes are involved in nutrient reservoir activity, DNA integration, glucan metabolism, carbohydrate metabolism, and protein process metabolism. At 21 DPA, the enriched genes are involved in nutrient reservoir activity, ribosome, amino acids metabolism, translation, protein process metabolism, etc. At 28 DPA, enriched genes are mainly involved in ribosome, amino acids metabolism, translation, and protein process metabolism (Figure 1 and Supplementary Figure S2).

In total, 621 proteins were differentially expressed (Supplementary Table S2). Among the four different stages, more DEPs were observed at 7 and 28 DPA (232 and 228) compared with 14 and 21 DPA (110 and 144) (Table 1). The Venn diagram of DEPs at different stages (Supplementary Figure S1) revealed that 13 DEPs were repeatedly detected at both 7 and 14 DPA, 37 DEPs were repeatedly detected at both 21 and 28 DPA. Only two proteins were differentially expressed at all four stages, they are TraesCS3A02G027800, which is a transcription initiation factor IIE subunit beta and TraesCS4D02G343400, which is an alcohol dehydrogenase-like protein. Comparing the DEGs and DEPs, 3, 4, 7, and 13 common genes were found at 7, 14, 21, and 28 DPA (in total 20 genes because of the existence of overlaps), respectively (Table 2). Among them, gene TraesCS7B02G038200 and gene TraesCSU02G117700 were repeatedly detected in 14, 21, and 28 DPA. Gene TraesCS4A02G036200 was detected at both 7 and 14 DPA.

The GO analysis of DEPs suggested that, at all four developmental stages, the commonly enriched genes were characteristic with binding and catalytic activity and participated in cellular and metabolic processes (Supplementary Figure S3). The KEGG analysis of DEPs (Figure 1 and Supplementary Figure S2) suggested that at all four stages, the commonly enriched genes are involved in carbohydrate biosynthesis process, transcription, translation, protein process metabolism, amino acids metabolism, and lipid metabolism. Even though only a small proportion of common genes were differentially expressed, GO and KEGG analysis indicated that both DEGs and DEPs at different stages can be categorized into similar groups, namely carbohydrate biosynthesis process, transcription, translation, protein process metabolism, amino acids metabolism, and lipid metabolism (Figure 1, Supplementary Figures S2 and S3).

### 3.2. Accumulation Patterns of Different SSPs Varied between the Transgenic and Non-Transgenic Line

**Figure 2:**
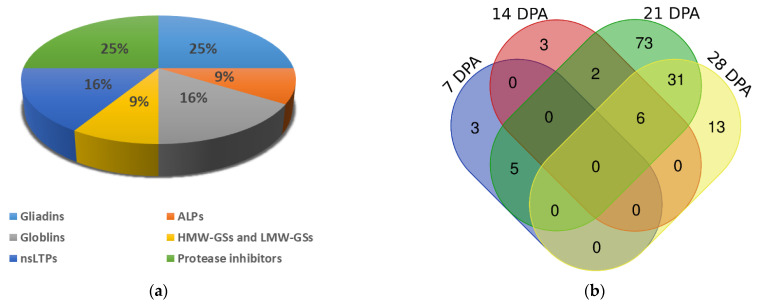
Composition of differentially expressed SSPs and expression patterns of different types of SSPs. (**a**) Composition of differentially expressed SSPs; (**b**) Venn diagram of differentially expressed SSPs at different seed developmental stages.

A total of 136 storage proteins were differentially expressed between the transgenic and non-transgenic lines at four stages, including glutenins, gliadins, nsLTP family, ALPs and PIs (Figure 2a). The most abundant SSPs were gliadins and PIs, followed by nsLTPs and globulins. The least abundant SSPs were ALPs and glutenins. Most of the SSPs were differentially expressed at 21 DPA, with 117 SSPs being differentially expressed at this stage (Figure 2b). Among those SSPs, five were also identified at 7 DPA that belong to the nsLTP family. Two were also identified at 14 DPA, including a gamma-gliadin TraesCS1D02G001000 and an ALP TraesCS7D02G031700. A total of 31 were also identified at 28 DPA, including 4 gliadin proteins, 5 ALPs, 4 globulins, 17 PIs, and 1 glutenin protein. The five HMW-GS genes (TraesCS1A02G317311, TraesCS1B02G329711, TraesCS1B02G329992, TraesCS1D02G317211, and TraesCS1D02G317301) and one ALP gene (TraesCS4A02G453405) were found differentially expressed between the transgenic and non-transgenic lines at 14, 21, and 28 DPA stages.

Different types of SSPs showed different expression patterns in the transgenic and non-transgenic lines at different stages (Table 3). At 7 DPA, a decrease in the expression of LTP family genes was observed in the transgenic line. At 14 DPA, the transgenic line displayed an increase in the expression of gliadins and ALPs and a decrease in glutenins and LTPs. At 21 DPA, the expression of all gliadins, all globulins, 1 ALP, 4 glutenins, 13 LTPs and 19 PIs increased in the transgenic line, while the expression of 10 ALPs, 6 glutenins, and 5 LTPs decreased. At 28 DPA, 5 gliadins, 5 globulins, 1 LTP, and 18 PIs were upregulated in the transgenic line, whereas 2 gliadins, 6 ALPs, 3 globulins, 7 glutenins, and 3 PIs were downregulated. It can be concluded that the significant decrease in HMW-GS expression was accompanied by the synergetic changes in expressions of gliadins, globulins, ALPs, LTPs, and PIs.

Compared with SSPs detected in DEGs, fewer SSPs were detected in DEPs, only 5, 3, 9, and 10 SSPs at 7, 14, 21, and 28 DPA, respectively (Table 4). Due to the constraints of detection method of DIA used in the current study, the gluten proteins including gliadins, HMW-GSs, and LWM-GSs were not detected in the proteome analysis. The SSPs detected were mostly PIs, LTPs, and globulins. Most of the SSPs were found downregulated, except some of the LTPs and globulins.

Three SSPs (TraesCS5D02G004000, TraesCS4A02G453600, and TraesCS5A02G424800) were commonly detected using both transcriptome and proteome analyses. They were all detected at 28 DPA and were downregulated in both analyses.

### 3.3. Comparison of Starch and Sucrose Metabolism-Related DEGs and DEPs

A total of 61 starch and sucrose metabolism-related genes were found differentially expressed between the transgenic and non-transgenic line (Supplementary Table S4). Several genes among them were repeatedly detected at multiple stages. For example, TraesCS2B02G240100 (beta-amylase) was downregulated at both 7 and 14 DPA. TraesCS7D02G535400 (1,4-alpha-glucan branching enzyme) was upregulated at 14 DPA and downregulated at 28 DPA. TraesCS7D02G036600 (sucrose synthase) was upregulated at both 7 and 14 DPA. TraesCS2B02G157800 (beta-glucosidase 26) was upregulated, while TraesCS3B02G530500 (glucan endo-1,3-beta-glucosidase) and TraesCS5D02G398900 (beta-glucosidase) were downregulated at 21 and 28 DPA. Another beta-glucosidase gene TraesCS5B02G393900 was upregulated at 21 DPA and downregulated at 28 DPA. Its homologous gene TraesCS5A02G388300 was upregulated at 14 DPA. Similar to SSPs, starch-related DEGs were also the most abundant at 21 DPA. Among the 43 DEGs detected at 21 DPA, 7 belong to wheat starch branching enzyme genes, all were downregulated.

**Figure 3:**
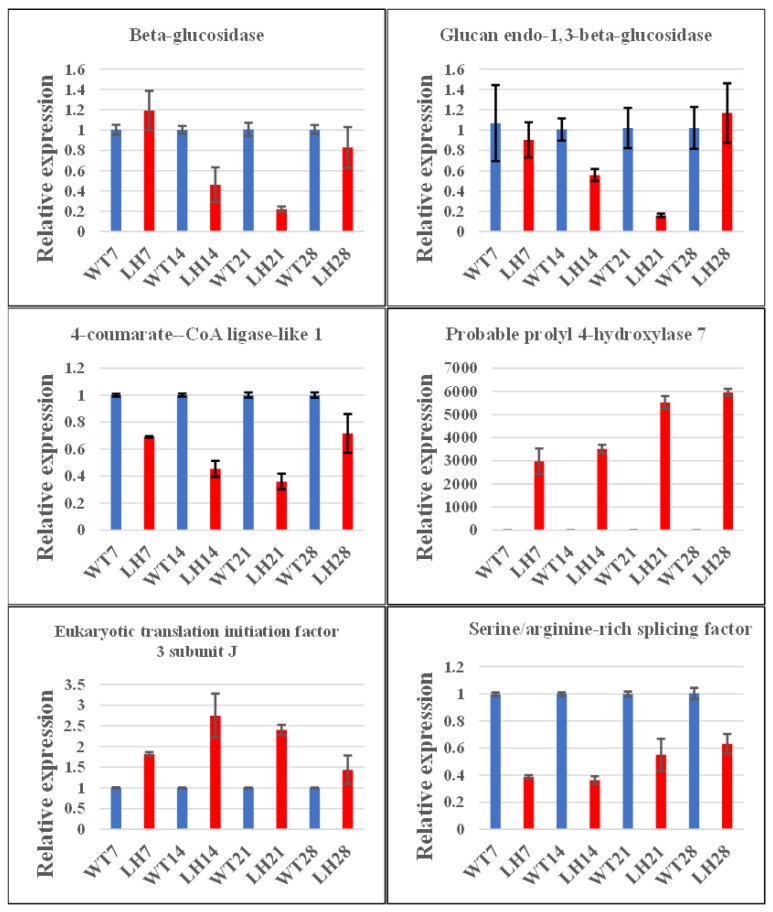
Relative expression of key genes at four seed developmental stages. Blue bar represents the wildtype (WT), red bar represents LH-11; expression level of WT was standardized at each stage to display a clear comparison with LH-11.

A total of 69 DEPs were categorized as starch and sucrose metabolism-related according to KEGG and GO classification (Supplementary Table S5). Among them, 22, 11, 13, and 28 were expressed at 7, 14, 21, and 28 DPA, respectively. Most of these DEPs were downregulated, significantly upregulated DEPs were TraesCS2B02G424300 (sucrose synthase 7) and TraesCS7B02G139700 (isoamylase 1) at 7 DPA, TraesCS6A02G302400 (2,3-bisphosphoglycerate) at 14 DPA, TraesCS2A02G109600 (pectinesterase 1) and TraesCS7A02G075600 (enolase 1) at 21 DPA, and TraesCS2A02G233500 (plant mobile domain protein) at 28 DPA. It is also worth noting that homologous genes were synergically regulated. For example, TraesCS3B02G530500 and TraesCS3D02G478800, TraesCS3B02G530600, and TraesCS3D02G478900, all are glucan endo-1,3-beta-glucosidase GIV and were downregulated at 7 DPA. TraesCS3A02G260100 and TraesCS3D02G260300 are 26 kDa endochitinase 1 were both downregulated at 28 DPA. The RT-PCR results of gene TraesCS3B02G530500 and TraesCS5D02G398900 confirmed the downregulation of these two beta-glucosidase genes in the transgenic line, especially at 21 DPA (Figure 3).

More similarities between the transcriptome and proteome could be found if the homologous genes were taken into consideration. Gene TraesCS2B02G240100 is a beta-amylase, which was downregulated at 7 DPA in transcriptome, and one of its homologous gene TraesCS2D02G220900 was also downregulated at 7 DPA in the proteome. Gene TraesCS2B02G157800 is a beta-glucosidase 26, which was upregulated at 21 DPA in transcriptome, and one of its homologous gene TraesCS2A02G134300 was also upregulated at 21 DPA in proteome. Gene TraesCS7D02G535500 is a 1,4-alpha-glucan-branching enzyme, as both a DEG and DEP, it was downregulated at 21 DPA. Other 1,4-alpha-glucan-branching enzyme DEGs (TraesCS2A02G293400, TraesCS2A02G310300, TraesCS2D02G290800, TraesCS2B02G309500, TraesCS2D02G308600 and TraesCS7A02G549300) were also found downregulated. TraesCS6A02G093200 and TraesCS6B02G122000 as DEGs and TraesCS1A02G072300 as a DEP were found upregulated, all belonging to endoglucanase protein family.

### 3.4. Comparisons of Amino Acids Biosynthesis-Related DEGs and DEPs

In total, 96 genes related to amino acids biosynthesis were differentially expressed at four stages between the two lines (Supplementary Table S6). Among them, gene TraesCS4A02G036200, which is a 4-coumarate-CoA ligase-like 1 protein, participates in the phenylalanine metabolism pathway and TraesCS4A02G036400, which is D-tagatose-1,6-bisphosphate aldolase subunit, participates in the phenylalanine and tyrosine metabolism pathway, were significantly downregulated at all four stages. TraesCS5A02G427800 was upregulated at 7, 14, and 21 DPA, which is an indole-3-acetaldehyde oxidase, involved in the tryptophan pathway. Another indole-3-acetaldehyde oxidase gene TraesCS5A02G427600 was downregulated at 7 and 14 DPA. Gene TraesCS5A02G612300LC, a probable prolyl 4-hydroxylase 7, was significantly upregulated at 7, 14, and 28 DPA, gene BGI_novel_G010394 was upregulated at 14 and 28 DPA, which is a dUTP pyrophosphatase.

A total of 42 DEPs were found involved in amino acids metabolism (Supplementary Table S7). Most of the DEPs identified were downregulated. Significantly regulated DEPs included TraesCS1A02G170900 (probable prolyl 4-hydroxylase 3), which was upregulated at 7 DPA. Another probable prolyl 4-hydroxylase, TraesCS5B02G459100, was also upregulated at 7 and 14 DPA. TraesCS4A02G262600 and TraesCS3A02G022600, belonging to glutamate decarboxylase, were downregulated at 7 and 28 DPA, respectively.

TraesCS4A02G036200 (4-coumarate-CoA ligase-like 1) was downregulated as a DEG at all four stages, while as a DEP, it was downregulated at both 7 and 14 DPA. Gene TraesCS5A02G612300LC (probable prolyl 4-hydroxylase 7) was significantly upregulated at 7, 14, and 28 DPA. Similarly, prolyl 4-hydroxylase-related DEPs TraesCS1A02G170900 and TraesCS5B02G459100 were also upregulated. Glutamate dehydrogenase genes BGI_novel_G001695 and BGI_novel_G001876 in DEG and glutamate decarboxylase gene TraesCS4A02G262600 and TraesCS3A02G022600 in DEP were all downregulated. TraesCS4B02G047400, TraesCS4D02G047400, and TraesCS6D02G065600 in DEG, TraesCS1D02G141800 and TraesCS4A02G266900 are Glutamine synthetase, were found up- and downregulated. The RT-PCR results (Figure 3) confirmed the downregulation of TraesCS4A02G036200 (4-coumarate-CoA ligase-like 1) and the upregulation of TraesCS5A02G612300LC (probable prolyl 4-hydroxylase 7) in the transgenic line.

### 3.5. Comparisons of Transcription, Translation, and Protein Processing-Related DEGs and DEPs

There were 365 DEGs in this category being identified between the transgenic and non-transgenic lines at four stages (Supplementary Table S8). Those DEGs were associated with RNA transport, degradation and surveillance, protein processing, splicesome, RNA polymerase. Gene BGI_novel_G003657 (DNA repair protein REV1/translation factor GUF1) and TraesCSU02G117600 (eukaryotic translation initiation factor 3 subunit J) were upregulated at all four stages. TraesCSU02G119700 (serine/arginine-rich splicing factor RS41), TraesCS2B02G406400 (GATA transcription factor) and BGI_novel_G010402 (MYB domain-containing protein-like) were downregulated at all four stages. BGI_novel_G000075 (DNA repair protein REV1/translation factor GUF1) was upregulated at 7, 14, and 28 DPA. TraesCSU02G117800 was upregulated at 7, 14, and 21 DPA. Those DEGs can be categorized into several protein families, including heat shock 70 kDa protein 4, glycine-rich RNA-binding protein, serine/arginine-rich splicing factor, DEAD-box ATP-dependent RNA helicase, elongation factor, eukaryotic translation initiation factor, RNA polymerase, etc. Downregulated DEGs include splicing factors, translation initiation factors, GATA transcription factor, and NAC domain protein. Heat shock proteins (HSPs), participating in protein process metabolism, were found upregulated at all four stages, RNA helicase related DEGs were all found downregulated.

At 7 DPA, transcription, translation, and protein processing-related DEPs were all found downregulated. Heat shock proteins were upregulated at 14 DPA (Supplementary Table S9). Glycine-rich RNA-binding proteins in both DEP (TraesCS3D02G417800 and TraesCS3B02G457900) and DEG (TraesCS4A02G293000 and TraesCS4B02G020300) were found downregulated. Downregulation was also found for serine/arginine-rich splicing factor in DEP (TraesCS3A02G500500) and DEG (TraesCSU02G119700), translation initiation factor in DEP (TraesCS2B02G235100) and DEG (BGI_novel_G008988) and nuclear pore complex protein in DEP (TraesCS4D02G239000 and TraesCS2A02G246000) and DEG (BGI_novel_G010402, BGI_novel_G010772 and BGI_novel_G010403). Upregulation was found for pentatricopeptide repeat (PPR) family genes in both DEP (TraesCS5A02G467500) and DEG (BGI_novel_G009861). At 14 DPA, downregulation for pre-mRNA-splicing factor was found in both DEP (TraesCS5A02G159100) and DEG (TraesCSU02G119700), while upregulation was found for HSPs in DEP (TraesCS4A02G092900, TraesCS4D02G243000, and TraesCS4B02G397600) and DEG (TraesCS7A02G177700, TraesCS1A02G285000, and TraesCS1D02G284000), peptide chain release factor in DEP (TraesCS1B02G098300) and DEG (TraesCSU02G051800), translation initiation factor proteins in DEP (TraesCS6A02G066400 and TraesCS1A02G331200) and DEG (TraesCSU02G117600). At 21 DPA, downregulation for ubiquitin-protein ligase was found in both DEP (TraesCS7D02G000400) and DEG (TraesCSU02G139900), glycine-rich RNA-binding protein in DEP (TraesCS4B02G020300) and DEG (TraesCS4D02G018500). At 28 DPA, down-regulation for serine/arginine-rich splicing factor was found in DEP (TraesCS7A02G569700) and DEG (TraesCSU02G119700), nuclear pore complex proteins in DEP (TraesCS6A02G175000) and DEG (TraesCS5A02G426400 and BGI_novel_G006757). Upregulation for 17.9 kDa class I HSP TraesCS4A02G092600 was found in both transcriptome and proteome. The upregulation of translation initiation factor (TraesCSU02G117600) and the downregulation serine/arginine-rich splicing factor (TraesCSU02G119700) were consistent with the RT-PCR results (Figure 3).

## 4. Discussion

### 4.1. Absence of HMW-GSs Promotes the Accumulation of Gliadins and Certain Protease Inhibitors

Major components of a wheat grain include starch, storage proteins, and lipids, which accounts for 60–70%, 8–15%, and 2.1–3.3% of total dry grain weight, respectively [40]. Various physiological and biochemical processes occur during the wheat grain development. In the first two weeks after anthesis, genes related to cytoskeleton and structure, DNA repair and replication, and cellular metabolism were found to have higher expression levels. Heat shock proteins were also expressed at early developmental stages. Starch and storage proteins are synthesized mainly at the grain filling stage at 14–28 DPA. In this process, genes belong to pathways involved in amino acid biosynthesis, carbohydrates metabolism are generally active [41,42].

The prolamin superfamily include glutenins, gliadins, farinins, purinins, puroindolines (Pins), GSPs, LTPs, ATIs, and thionins. Farinins and purinins also known as b-type ALPs and a type of LMW-GSs, respectively. Both contribute positively to dough quality [14]. They are categorized as globulins based on solubility. Avenin-like proteins, GSPs, ATIs, and several types of gliadins share the same protein domains PF13016 and PF00234. According to their water- and salt-soluble properties, these proteins can be categorized into albumins and globulins [43]. Different types of SSPs possess different functions. Grain softness proteins may contribute to hardness, while many LTPs and ATIs are characterized as wheat allergens. Germins are globulins that involve in cell responses to desiccation, dehydration, and osmotic stress [44]. Thionins, ribosome inactivating proteins, and other defense-related proteins present in wheat grains are mostly globular proteins that are tightly integrated by multiple interchain disulphide bonds with high stability [45]. In the current study, apart from the silent HMW-GS coding genes, other SSP genes such as gliadins, ALPs, GSPs, ATIs, LTPs, and Pins also showed differential expression between the transgenic and non-transgenic line. This type of synergetic and simultaneous change of SSP profile was also found in other transgenic lines when one prolamin component was altered [46]. It suggests that the change of a major component of the SSPs also affects the accumulations of other SSP constituents, mainly due to the similarities of protein structures shared by the SSPs. Different SSP constituents distinctly contribute to wheat end-use quality and have different immunogenic properties. Thus, many attempts have been put forward to alter or remove certain types of SSPs [18,46,47,48]. However, this co-migration or simultaneous change in the unintended SSPs adds a complexity in wheat breeding for quality improvement. Therefore, a better understanding of the regulation mechanism under different SSPs is crucial for targeted improvement of certain SSP constituents.

### 4.2. Transcriptome and Proteome Studies-Related to SSPs

Omics techniques have been widely applied in wheat SSP studies [36,40,41,49,50,51,52,53,54,55,56]. In particular, proteomics [16,17] and transcriptomics [18] were used to compare the differences between non-transgenic and SSP transgenic lines.

Up to now, wheat grain proteomics on various cultivars have been extensively carried out [2,8,17,19,53,56,57,58,59,60]. One proteomics study on the transgenic lines in which certain gliadins were absent indicated that co-migration exists between gliadins and other proteins [46]. Hence, it is possible to use proteomics to unravel the regulation networks of storage proteins by study the storage protein transgenic lines.

A low consistency (38.9%) between gene transcription and protein expression is often observed [61]. Similar results were also obtained in the current study, among the 2962 DEGs and 621 DEPs, only 20 common genes were identified. The timing differences in gene transcription, protein translation and post-translational modifications might be the cause of this inconsistency.

### 4.3. Coding Genes for Glutenin Genes and Transgenic Study on HMW-GS

Glutenin proteins include the HMW glutenins and LMW glutenins. Wheat HMW-GSs coding genes *Glu-A1*, *Glu-B1* and *Glu-D1* are located on chromosome 1A, 1B, and 1D, designated as the *Glu-1* locus, which was found to control protein quality by balancing the ratios of HMW/LMW and glutenins/gliadins and consequently the formation of GMP [62]. Even though only accounting for 10% of the total storage proteins in wheat, HMW glutenins are considered the major determinants of wheat quality. Allelic variations at *Glu-B1* and *Glu-D1* both have significant effects on protein quality [6]. Typical LMW-GSs are encoded by the *Glu-3* loci, located on the short arms of homoeologous group-1 and tightly linked to the *Gli-1* homoeoloci [63,64]. Different LMW-GS loci allelic variations contribute differently to wheat protein quality. The *Glu-A3b* and *Glu-A3d* at *Glu-A3* locus, the *Glu-B3b*, *Glu-B3g,* and *Glu-B3h* at *Glu-B3* locus and the *Glu-D3b* and *Glu-D3a* at *Glu-D3* locus are considered superior LMW-GS alleles, which all contribute positively to wheat end-product quality [3,65]. However, the lack of information for each allele score limits the utilization of LMW-GS optimization for quality improvement. Seven LMW-GS genes belonging to *Glu-A3* and *Glu-D3* loci were differentially expressed between the two lines in this study. For *Glu-A3* genes, TraesCS1A02G010900 was downregulated at 7 DPA, TraesCS1A02G007934 and TraesCS1A02G010905 were upregulated at 14 DPA. For *Glu-D3* genes, TraesCS1D02G007400 was downregulated at 21 and 28 DPA, TraesCS1D02G008600 and TraesCS1D02G009900 were upregulated at 21 DPA, while TraesCS1D02G015100 was downregulated at 28 DPA. Results from this study suggested a common regulation mechanism was shared by the HMW-GS and LMW-GS coding genes. Hence, the manipulation of one type of glutenin might affect the accumulation of other glutenins.

Due to the importance of prolamin proteins in grain quality determination, multiple attempts have been made to study and evaluate the effects of different prolamin constituents on wheat protein quality through transgenic approaches [5,16,17,18,48,62,66,67,68]. Over-expression of additional HMW-GS coding genes can lead to changes in dough strength and gluten protein composition [5,48,66,67,68]. Introgression of the commonly silent *Glu-1Ay* was found to have positive effects on wheat quality [69]. Similar results were found for introgression of *1E*-encoded storage protein from *Agropyron elongatum* into Chinese Spring [70]. Improvement in dough strength-related parameters, including the dough stability and peak time of Farinograph, mixing time of Mixograph, etc. was observed when an extra *Dy* gene was introduced [5]. These changes are probably caused by the alterations in the secondary and micro-structures of the gluten network [68]. The absence of HMW-GS *Ax1* or *Dx2* decreased the accumulation of gluten polymers, which lead to the decrease in the dough development time and stability [71], while the absence of *1Dy12* was considered to stimulate the accumulations of gliadins and LMW-GSs [62]. In addition to the effects on prolamin proteins, other proteins including enzymes related to carbohydrates metabolism were also affected in prolamin gene transgenic lines [46].

In the current study, the change of HMW-GS expression in the transgenic line triggered a series of changes in the expression levels of other genes. The most enriched DEGs participated in amino acids metabolism and protein process metabolism, reflected by the GO and KEGG analyses. In addition, at 7 and 14 DPA, genes involved in lipid and carbohydrates metabolisms were also altered. Similar results were observed through the proteomics study. At all four stages, enriched proteins were involved in carbohydrate biosynthesis process, transcription, translation, protein process metabolism, amino acids metabolism, and lipid metabolism, which suggested a crosstalk between the protein biosynthesis and carbohydrates metabolism.

### 4.4. TGS and PTGS and Their Underlying Mechanisms

In bombardment-treated transgenic plants, the integration position of the transgene is unknown. This integration could damage other genes by interrupting gene function directly. It can also cause the simultaneous co-suppression of other endogenous genes [72]. In this case, the genes other than the transgene between the transgenic and non-transgenic line were differentially expressed. Transcriptional gene silencing (TGS) and post-transcriptional gene silencing (PTGS) are the two common mechanisms underlying the co-suppression phenomenon in transgenic plants [73].

Two pathways are considered important by accumulated results in silencing transgenes and transposon elements (TEs) by organisms. First, on TGS level, methylation and/or chromatin configuration has a role in suppressing the transcription of TEs. Second, on PTGS level, transcripts of TEs are degraded. These pathways are speculated to act independently or synergistically to silence plant TEs. Homologous DNA, once being introduced in plants, can cause DNA–DNA pairing, which has been reported to trigger TGS. Polycomb (PC) proteins were reported to mediate such DNA–DNA interactions [74]. Transgene can undergo TGS when integrated at or near a hypermethylated region resembles position effect variegation (PEV). Local heterochromatin formation and silencing of neighboring genes can be induced by the extension of transgene repeats. Such repeat-induced gene silencing in plants possibly correlates with changes in chromatin configuration [73]. Genes that are highly expressed during grain development are more susceptible to transgene-caused damage as they are in open chromatin status for the accumulation of large quantities of various SSPs. Post-transcriptional gene silencing can silence multiple homologous genes at the same time, which is an important regulation mechanism, especially for polypoid plants. The existence of repeat sequences are key in the induction of endogenous PTGS [75]. The RNA recognition motif (RRM), which is present in RNA-binding proteins (RBPs) such as splicing factors, plays an important role in the sequence specificity during the PTGS process [73]. Overexpression of RBPs enhances PTGS, RBPs promote PTGS by facilitating siRNA accumulation and compromising RNA silencing suppression [76]. In the current study, downregulation of multiple genes containing RRM domain was identified in both DEGs and DEPs, including splicing factors, glycine rich proteins, *XRN4*, ribosomal proteins (RPs), RNA binding proteins, ATP-dependent RNA helicase. This downregulation of RBPs in the transgenic line suggests that the silence of HMW-GS genes is controlled on the TGS level, and the above RBPs are key genes participated in glutenin genes biosynthesis.

In response to an environmental stimulus such as exogenous gene integration, the genome undergoes epigenetic modifications, including chromatin reconstruction, methylation, etc. [77]. To prevent proliferation of inserted sequences, silencing of near genes is common to prevent the production of aberrant transcripts via read-through transcription [78,79]. The neighboring genes of inserted sequences can also be affected through splicing and polyadenylation patterns alteration. In Arabidopsis, it was found that gene *IBM1* counteracts DNA methylation of endogenous genes while *JMJ14* acts on expressed transgenes, which caused by the different epigenetic features between the transgene and endogenous genes [80]. The HMW-GS coding genes contain relatively higher GC content. Therefore, the insertion of HMW-GS sequence could cause changes in epigenetic feature, including methylation and/or chromatin rearrangements. Learning more about the silencing mechanism in this important example can guide potential positive applications of gene silencing. It could be potentially used in silencing allergic proteins in wheat.

### 4.5. Carbon and Nitrogen Metabolism

Wheat grains are the major sink organs, predominant genes involved in the grain development are associated with nutrient reserves, carbohydrates metabolism, and plant defense [40]. The accumulation of SSPs is source limited while the starch accumulation is sink limited, the tight links between carbon and nitrogen metabolism and the equilibrium of N/C ratio greatly influence the abundance of the SSPs.

- Nitrogen

Seed storage proteins are firstly formed at the cytoplasmic side of the rough endoplasmatic reticulum (ER), they are then translocated to the endomembrane system with the aid of N-terminal peptides. After biosynthesis, they are translocated to the ER lumen where protein modifications occur. Proteins related to nitrogen metabolism display a decrease trend during grain filling [61], which is consistent with the trend of SSP deposition during grain filling and maturation. Both DEGs and DEPs were enriched in nitrogen metabolism revealed by GO and KEGG analyses, which indicated that the silence of HMW-GSs in the transgenic line leads to various changes in the nitrogen metabolism related genes.

Of the nine essential amino acids, lysine (Lys), methionine (Met), threonine (Thr), and isoleucine (Ile) are produced from aspartate (Asp) via a branched and complicated pathway, and are commonly known as Asp-family amino acids [81]. Gluten proteins are rich in glutamine and proline, which comprise from 43% to 73% of the total amino acids [82]. High expression levels of nitrogen metabolism enzymes encoding genes including *TaGS*, *TaAlaAT*, and *TaWCP2* contributed to the rapid synthesis of glutenins, which resulted in higher GMP content [83]. At 21 DPA, a decreased accumulation of glutamine and glycine was found in LH-11, which could be reflected by the downregulation of glutamine synthetase (TraesCS6D02G065600) and betaine aldehyde dehydrogenase (TraesCS2B02G346000), respectively. At 28 DPA, a decrease of tryptophan and increase of methionine and glutamine was found in LH-11, which could be reflected by the downregulation of tryptophan synthase (BGI_novel_G010390) and upregulation of homocysteine S-methyltransferase (TraesCS4B02G242700) and glutamine synthetase (TraesCS4B02G047400). The differential expression of glutamine synthesis-related genes and upregulation of prolyl 4-hydroxylase genes which participate in proline catabolism is consistent with the differential deposition of prolamin proteins between the transgenic and non-transgenic line used in the current study.

- Carbon

As the predominant composition of carbohydrates, starch is synthesized by the coordinated reactions of four main enzymes, i.e., adenosine diphosphate ADP pyrophosphorylase (ADP-Glc PPase), starch synthase (SS), starch branching enzyme (SBE), and starch debranching enzyme (DBE). Starch formation in wheat grains requires the import of sugar in the form of sucrose from the source organs. Sucrose can upregulate the expression of enzymes such as Sucrose synthase (SuSy) and ADP-Glc pyrophosphorylase (AGPase), and induce storage-associated gene expression at the transcript level [84,85]. Once in the grains, this sucrose is hydrolyzed by SuSy and invertases (INVs) into monomer sugars, glucose/UDP-glucose and fructose that are converted into glucose-1-phosphate, which is considered as the most efficient starch synthesis precursor. Glucose-1-phosphate is further converted to ADP-glucose and pyrophosphate by the action of AGPase. ADP-glucose acts as a substrate for the biosynthesis of the two types of starch, amylose, and amylopectin. The formation of amylose is mediated by granule bound starch synthase (GBSS) while that of amylopectin is catalyzed by the combined action of SS, SBE, and DBE [86]. Starch synthase genes in plants are encoded by several genes including SSI, SSII, SSIII, and SSIV. Individual members of these gene families are believed to have specific roles in the formation of amylopectin [87]. During seed development, genes such as UDP glucose-6-dehydrogenase, starch phosphorylase, Susy, 1,4-alpha-glucan branching enzyme, starch synthase, fructokinase, endoglucanase, glucose-6-phosphate isomerase, trehalose 6-phosphate synthase/phosphatase, and ADP-sugar diphosphatase were found differentially expressed between different stages [41].

Amylose and amylopectin are the two main components of starch. Slight upregulation of GBSS at 14 DPA and downregulation of SS at 21 DPA in the current study suggest changes in starch composition may occur between the two lines, the absence of HMW-GS is accompanied by a decrease of amylopectin in starch composition.

Beta-glucosidase is responsible for the catalyzation of UDP-glucose into glucose, it is considered as one of the most abundant enzymes in wheat [40]. Beta-glucosidase related genes were both up- and downregulated at 21 and 28 DPA. This suggests, at the later stages of grain development, more activities occur in wheat grains from LH-11. Thus, more starch synthesis and catabolism are required to generate more energy (ATP) to satisfy additional metabolic activities.

Beta-amylase is a starch-degrading enzyme that hydrolytically cleaves α-1,4-D-glucosidic bonds, and is one of the major proteins in the starchy endosperm [88,89]. They can only contribute to starch granule hydrolysis by degrading solubilized intermediates that are released from the granules by α-amylase [58]. A positive correlation has been found between β-amylase activity and gliadins or salt-extractable proteins [90]. Downregulation of β-amylase was observed at 7 and 14 DPA in the transgenic line, suggesting a difference in grain development between the two lines. Therefore, a different amount of energy was produced during the early stages of grain development.

Protease inhibitors are abundantly expressed in the wheat grain. Similar to glutenins and gliadins, they tend to form protein complexes to prevent degradations of SSP substances. Among them, ATIs are bifunctional proteins that play important roles in preventing starch and proteins in the endosperm from degradation by blocking amylase and trypsin activities, especially under biotic and abiotic stresses [91]. Moreover, ATIs are responsible for the defense responses in wheat by blocking the enzyme activities of amylase in pests [40]. Serpins are involved in protein biosynthesis and degradation in wheat [58]. Upregulation of ATIs and Serpins in the transgenic line indicates a less degree of protein degradation in LH-11, which is desired in the baking process since they positively contribute to the breadmaking quality [12]. Downregulation of thionins from homologues group 1 and 3, known as the low molecular weight antimicrobial peptides, was found in the transgenic line, while upregulation of thionin genes from homologs group 5 was found at 21 and 28 DPA in the transgenic line.

### 4.6. Translation, Transcription, and Protein Process Metabolism

**Figure 4:**
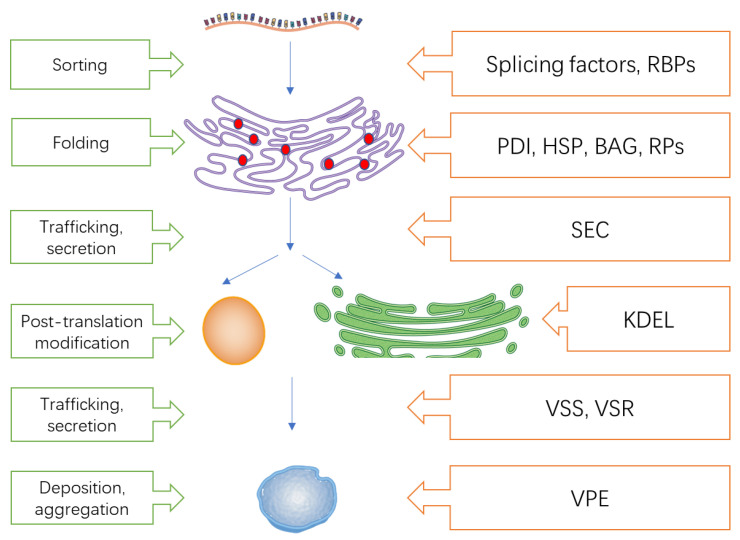
Important genes and proteins participated in the deposition of SSPs.

The mRNAs of SSPs are firstly sorted by RNA binding proteins (RBPs) before being translated into different proteins in ER. In the ER lumen, synthesized polypeptides are assembled and folded by enzymes such as protein disulphide isomerase (PDI) and chaperones before being sorted, secreted, and transported. Afterwards, mature acidic and the basic subunits of SSPs are generated by the precursor-accumulating (PAC) vesicles or Golgi, where post-translation modification of those SSPs occur. Mature proteins are deposited into the protein sorting vacuole (PSV)-derived protein bodies (PBs) or ER-derived PB in cereal grains. Vacuolar sorting signals (VSSs) and vacuolar sorting receptors (VSRs), are required for sorting SSPs [92]. Vacuolar protein sorting associated proteins (VSPs) are responsible for vacuolar protein trafficking and VSR recycling [93]. Vacuolar protein enzymes (VPE) and proteins with WD40 domains [94] promote the assembly and formation of large SSP complexes in PBs. Large numbers of proteins involved in this process showed differential expression in the current study (Figure 4), including genes coding RNA binding proteins, PDI, vacuolar sorting proteins, SEC proteins, protein transporters, protein chaperones, etc. Protein disulfide isomerase is involved in SSP targeting, it is responsible for assembling poly-peptides via disulfide bonds [95]. Slight upregulation of three PDI genes was found in the current study at 21 DPA in transcriptome.

Splicing factors and DEAD-box ATP-dependent RNA helicase are crucial factors affecting transcription initiation and alternative mRNA transcript splicing as well as biotic and abiotic stress tolerance mechanisms in plants [96,97,98]. Downregulation and upregulation of ATP-dependent RNA helicase at 21 and 28 DPA, respectively, were observed in the transgenic line. Different splicing factors were differentially regulated, almost all were downregulated in the transgenic line, expect for some that were upregulated at 28 DPA. The differential expressions of these two types of genes are possibly associated with the differential accumulation of different SSPs.

Glutathione S-transferase (GST) plays important roles in protecting cells from toxins and oxidative damage. WD-repeat-containing (WDR) proteins are involved in various molecular mechanisms including transcriptional mechanisms, RNA processing, signal transduction, and chromatin modification [99]. Both GSTs and WDR proteins are reported to regulate grain protein composition and seed development [100]. Twelve GST genes as DEGs and three GST genes as DEPs were found in the current study. The GST genes were downregulated at 7 DPA in both transcriptome and proteome, while at 21 and 28 DPA, GST genes were all upregulated in transcriptome and downregulated in proteome. Six DEGs and three DEPs containing WDR domains were identified in the current study, all were downregulated. Co-suppression of WDR containing genes in the HMW-GSs silenced line suggested their potential roles in HMW-GSs biosynthesis.

Results from the proteome in the current study revealed a number of differentially expressed RPs. As the major component for ribosome, RPs play crucial roles in the protein biosynthesis. Proteins RP 40s and RP 60s showed differential expressions under different nitrogen levels and were upregulated at 25–35 DPA at a high nitrogen level [101]. At 7 DPA, all RPs showed a downregulation in the transgenic line, whereas at other stages, both up- and downregulations were observed, and the majority of the genes were dowregulated.

Heat shock proteins are produced during seed maturation under various stress conditions, they can function as molecular chaperones. Heat shock protein family members HSP60s, HSP70s, and HSP90s participate in the protein folding process, which is achieved by forming stable complexes with folding intermediates of their protein substrates [58]. In addition, HSP20s are involved in protein aggregation protection and HSP100s in re-solubilization of protein aggregates [102]. It was reported that HSPA1_8, HSP20, HSP90s were upregulated at a medium nitrogen treatment, while HSP90B was upregulated at high nitrogen treatment [103]. In the current study, at 7 DPA, HSP20s and HSP70s were upregulated in transcriptome. At 14 and 28 DPA, HSP20s and HSP70s were all upregulated in the transgenic line in both transcriptome and proteome. At 21 DPA, both up- and downregulations were found for genes coding HSP20s and HSP70s in the transcriptome. These results suggest that HSPs play important roles in the accumulation of different SSPs, and HSP20s, in particular, may relate more with the accumulation of gliadins. The Bcl-2-related pathogenicity (BAG) family proteins are widely conserved among various organisms and function as complexes to assist HSP70 in protein folding. The BAG proteins in Arabidopsis confer abiotic resistance [102]. Upregulation of *BAG* genes was found in the late grain filling stage in wheat [101]. Upregulation of *BAG* genes in the transgenic line was found in transcriptome in the current study.

The SEC proteins are responsible for protein transport. Expression levels of different SEC proteins have been found to be under the influences of different nitrogen levels [103]. In the current study, Gene *SEC61*, mainly involved in protein secretion in ER, was upregulated, while *SEC23*, which controls the formation of transport vesicle, was downregulated at 21 DPA in the transgenic line in transcriptome. In proteome, SEC proteins were downregulated at 21 and 28 DPA in the transgenic line.

Compared with other metabolism pathways, this pathway contained the most significantly enriched genes in the current study, suggesting the difference in SSP accumulation is mainly regulated by genes from this pathway.

### 4.7. Transcription Factors That Are Involved in the Regulation of the Expression of SSPs

Promoter regions of HMW-GS coding sequences contain a series of conserved cis-acting elements, including Prolamin Binding Factor (PBF DOF) binding sites (TGCAAAG), double N-box bZIP binding sites (TGAGTCA), Skn-1 like motif (GTCAT), MYB binding sites AACA/TA motif, ABRE motif (ACGTGGC), RY core site element (CATGCA), and NF-YA (CBF or LEC1) binding sites (CCAAT) [23,24]. In the current study, 37 genes that belong to 18 types of TFs were differentially expressed in proteome and transcriptome data (Supplementary Table S10). Among them, upregulated TF families included MYB, bZIP, bHLH, NAC, WRKY, B3, and HSF. Three TFs were downregulated at all four stages in the transcriptome, of which TraesCS7B02G112300LC is a MYB TF, TraesCS2B02G406400 is a GATA TF, and TraesCS4A02G036000 is a E2F TF. The MYB TFs can bind to the AACA motif in the promoters of SSP genes in cereals including barley [30] and wheat [27]. Transcription factor MYB promotes the expression of both alpha-amylase and hor2 in barley. The NAC transcription factor superfamily members have also been widely reported to be involved in SSP regulation, e.g., NAC019 [104], NAC100 [105], and NAC77 [29]. In the current study, six MYB type TFs were significantly regulated, including TraesCS1A02G275800, TraesCS1B02G285000, TraesCS2B02G387800, TraesCS3D02G329400, TraesCS7B02G049000, and TraesCS7B02G049200. Seven NAC TFs TraesCS7A02G152400, TraesCS7A02G152500, TraesCS7A02G194700, TraesCS7A02G569300, TraesCS7B02G100300, TraesCS7D02G154200, and TraesCS7D02G196300 were all upregulated in the transgenic line, while another NAC TF TraesCS7B02G056300 was downregulated. Further validation of this gene on the regulation of HMW-GS is needed in the future.

## 5. Conclusions

To understand and clarify the influence of HMW-GSs deficiency on expression of wheat seed storage substances and regulation mechanism HMW-GSs, we compared a previously obtained transgenic line and its non-transgenic parent at different seed development stages on a transcriptomic and proteomic level. Results showed that differentially expressed genes and proteins were enriched in nutrient reservoir, starch metabolism, amino acid metabolism, and transcription, translation, and protein process metabolism. Co-migration of ALPs, ATIs, and LTPs was observed with the deletion of HMW-GS proteins. Moreover, an increase in the expression levels of gliadins were observed at 21 and 28 DPA in the transgenic line. Silence of the HMW-GSs also triggered changes in carbohydrate metabolism and starch component composition, which indicated the differential accumulation of SSPs caused a difference in energy supply between the two lines. Results from this study provide insights in unraveling the interaction network behind wheat storage substances accumulation. Multiple genes containing RRM domain, which participate in PTGS, were found downregulated in the transgenic line, indicating the silence of HMW-GS genes are regulated on a TGS level. According to the transcriptome and proteome data, a list of major genes involved in SSP accumulation was also proposed. Six genes from the MYB and eight genes from the NAC TF families are likely important regulators of HMW-GS accumulation. This is valuable information for clarifying the regulatory mechanism of HMW-GSs and accelerating wheat quality improvement breeding. However, further validations of their genetic functions are needed in future study.

## Figures and Tables

**Table 1:** Summary of differentially expressed genes and proteins between the transgenic and non-transgenic line at four stages.

Analysis	Developmental Stage	Upregulated	Downregulated	Total
Transcriptome	7 DPA	113	124	237
	14 DPA	108	90	198
	21 DPA	737	854	1591
	28 DPA	1088	256	1344
Proteome	7 DPA	46	186	232
	14 DPA	70	40	110
	21 DPA	64	80	144
	28 DPA	58	170	228

**Table 2:** Commonly identified genes from both transcriptome and proteome at different stages.

Stage	Gene ID	Gene Description
7 DPA	TraesCS4A02G036200	4-coumarate-CoA ligase family protein
	TraesCS2B02G292100	Aldo/keto reductase family protein
	TraesCS3B02G102100	Fatty acid oxidation complex subunit alpha
14 DPA	TraesCS4A02G036200	4-coumarate-CoA ligase family protein
	TraesCS7B02G038200	Chalcone-flavonone isomerase
	TraesCSU02G117700	DnaJ domain protein
	TraesCS2A02G566600	DDB1- and CUL4-associated factor homolog 1
21 DPA	TraesCS3B02G584700	Pathogenesis-related protein PR-4
	TraesCS7B02G038200	Chalcone-flavonone isomerase
	TraesCS4A02G130800	Ricin B-like lectin R40C1
	TraesCS7A02G036000	Transmembrane protein, putative
	TraesCS7D02G535500	1,4-alpha-glucan-branching enzyme
	TraesCSU02G117700	DnaJ domain protein
	TraesCS5D02G004000	Grain softness protein
28 DPA	TraesCS4A02G453600	Gliadin-like avenin
	TraesCS2B02G392500	Kunitz trypsin inhibitor
	TraesCS4B02G173700	Ricin B-like lectin R40C1
	TraesCS4A02G130800	Ricin B-like lectin R40C1
	TraesCS2B02G292100	Probable aldo-keto reductase 2
	TraesCS4A02G092900	Heat-shock protein
	TraesCSU02G117700	DnaJ domain protein
	TraesCS1A02G233200	Signal peptidase I
	TraesCS7A02G070900	Peroxidase
	TraesCS5A02G424800	Dehydrin
	TraesCS1B02G322900	Transcription elongation factor SPT6
	TraesCS7A02G325700	Seed maturation protein

**Table 3:** Expression trends of different types of SSPs at different stages.

	7 DPA		14 DPA		21 DPA		28 DPA	
	Upregulated	Downregulated	Upregulated	Downregulated	Upregulated	Downregulated	Upregulated	Downregulated
Gliadins	0	0	1	0	30	0	5	2
ALPs	0	0	3	0	1	10	0	6
Globlins	0	0	0	0	18	0	5	3
HMW-GSs and LMW-GSs	0	0	0	6	4	6	0	7
nsLTPs	0	6	0	1	13	5	1	0
Protease inhibitors	0	0	0	0	19	11	18	3

**Table 4:** Differentially expressed SSPs at different stages in the proteome.

Stage	Gene ID	Fold Change	Description
7 DPA	TraesCS1B02G059000	−1.21527	11S globulin seed storage protein 2
	TraesCS2A02G375400	−1.37033	Kunitz trypsin inhibitor
	TraesCS2B02G392500	−2.65509	Kunitz trypsin inhibitor
	TraesCS2D02G371800	−1.13515	Kunitz trypsin inhibitor
	TraesCS3D02G323200	−1.12579	Protease inhibitor/seed storage/lipid transfer protein family protein
14 DPA	TraesCS1A02G007700	−1.10619	Gamma gliadin
	TraesCS5A02G432100	1.535714	Globulin-1
	TraesCS4A02G135500	1.444786	Vicilin-like seed protein
21 DPA	TraesCS2B02G471000	1.595181	Protease inhibitor/seed storage/lipid transfer family protein
	TraesCS2D02G449000	1.386938	Protease inhibitor/seed storage/lipid transfer family protein
	TraesCS2D02G449100	1.127106	Protease inhibitor/seed storage/lipid transfer family protein
	TraesCS5A02G424800	−1.52425	Dehydrin
	TraesCS5B02G426700	−1.26968	Dehydrin
	TraesCS5B02G426800	−1.21027	Dehydrin
	TraesCS5D02G004000	−1.25508	Grain softness protein
	TraesCSU02G108500	−1.72641	Alpha-gliadin
	TraesCS4B02G393400	−1.16845	Lipid-transfer protein
28 DPA	TraesCS1D02G046400	−1.22112	11S globulin seed storage protein
	TraesCS3B02G062600	−1.92171	Non-specific lipid-transfer protein
	TraesCS5B02G145900	−1.28086	Non-specific lipid-transfer protein
	TraesCS5D02G004000	−1.34982	Grain softness protein
	TraesCS7D02G504800	−1.14673	Puroindoline b
	TraesCS2B02G392500	−1.23746	Kunitz trypsin inhibitor
	TraesCS3B02G515100	−1.37625	Basic 7S globulin
	TraesCS4A02G453600	−2.67785	Gliadin-like avenin
	TraesCS5D02G145300	−1.09181	Non-specific lipid-transfer protein
	TraesCS5A02G424800	−1.11499	Dehydrin

## Data Availability

The data presented in this study are available in the article and Supplementary Material.

## Supplementary Materials

The following supporting information can be downloaded at: https://www.mdpi.com/article/10.3390/foods12020361/s1. Figure S1: Venn diagrams of differentially expressed genes and proteins at four stages. a Results from the transcriptomics, b results from the Proteomics. Figure S2: GO and KEGG enrichment of DEGs at different stages. GO enrichment results at 7, 14, and 28 DPA are represented by a, c, and e, respectively. KEGG enrichment results at 7, 14, and 28 DPA are represented by b, d, and f, respectively. Figure S3: GO and KEGG enrichment of DEPs at different stages. a GO enrichment results of upregulated and downregulated DEPs at 7 DPA; b GO enrichment results of upregulated and downregulated DEPs at 14 DPA; c GO enrichment results of upregulated and downregulated DEPs at 21 DPA; d GO enrichment results of upregulated and downregulated DEPs at 28 DPA. Table S1: Detailed information of the total 2262 DEGs at four stages. Table S2: Detailed information of the total DEPs at four stages. Table S3: Descriptions of the 36 genes commonly identified at all four stages. Table S4: DEGs related to starch and sucrose metabolism at four stages. Table S5: Starch and sucrose metabolism related DEPs at different stages. Table S6: DEGs related to amino acid biosynthesis at different stages. Table S7: DEPs related to amino acid metabolism at different stages. Table S8: DEGs related to transcription, translation, and protein process at different stages. Table S9: DEPs related to transcription, translation, and protein process at different stages. Table S10: Transcription factors identified in transcriptome and proteome at different stages. Table S11: Primers used for RT-PCR.
