# Supplementary material for: Transcriptome and Proteome Analysis Revealed the Influence of High-Molecular-Weight Glutenin Subunits (HMW-GSs) Deficiency on Expression of Storage Substances and the Potential Regulatory Mechanism of HMW-GSs
Source: Foods. 2023 Jan 12;12(2):361. doi: 10.3390/foods12020361 (PMC9857648; doi:10.3390/foods12020361)
Supplement: Supplementary file 1 [file foods-12-00361-s001.zip › Supplementary Figues.pdf]

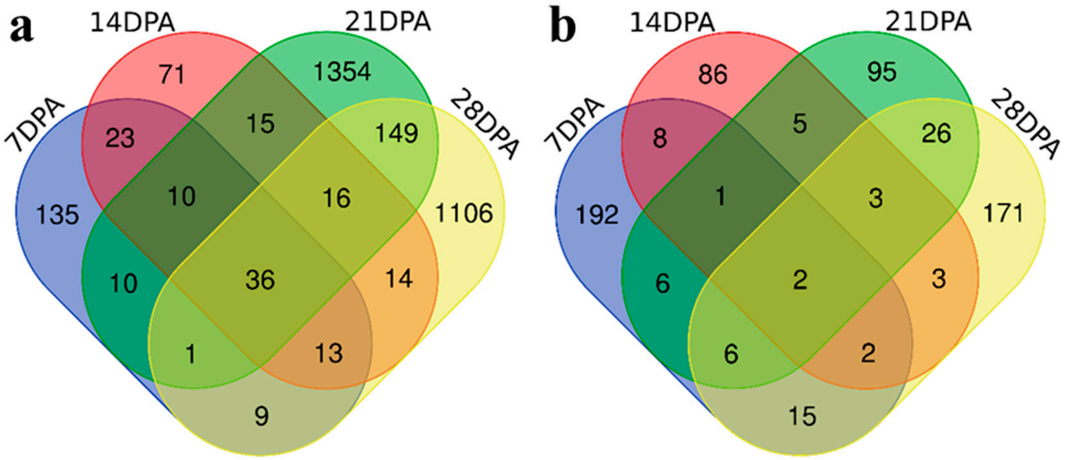

Figure S1 Venn diagrams of differentially expressed genes and proteins at four stages. a Results from the transcriptomics, b results from the Proteomics.

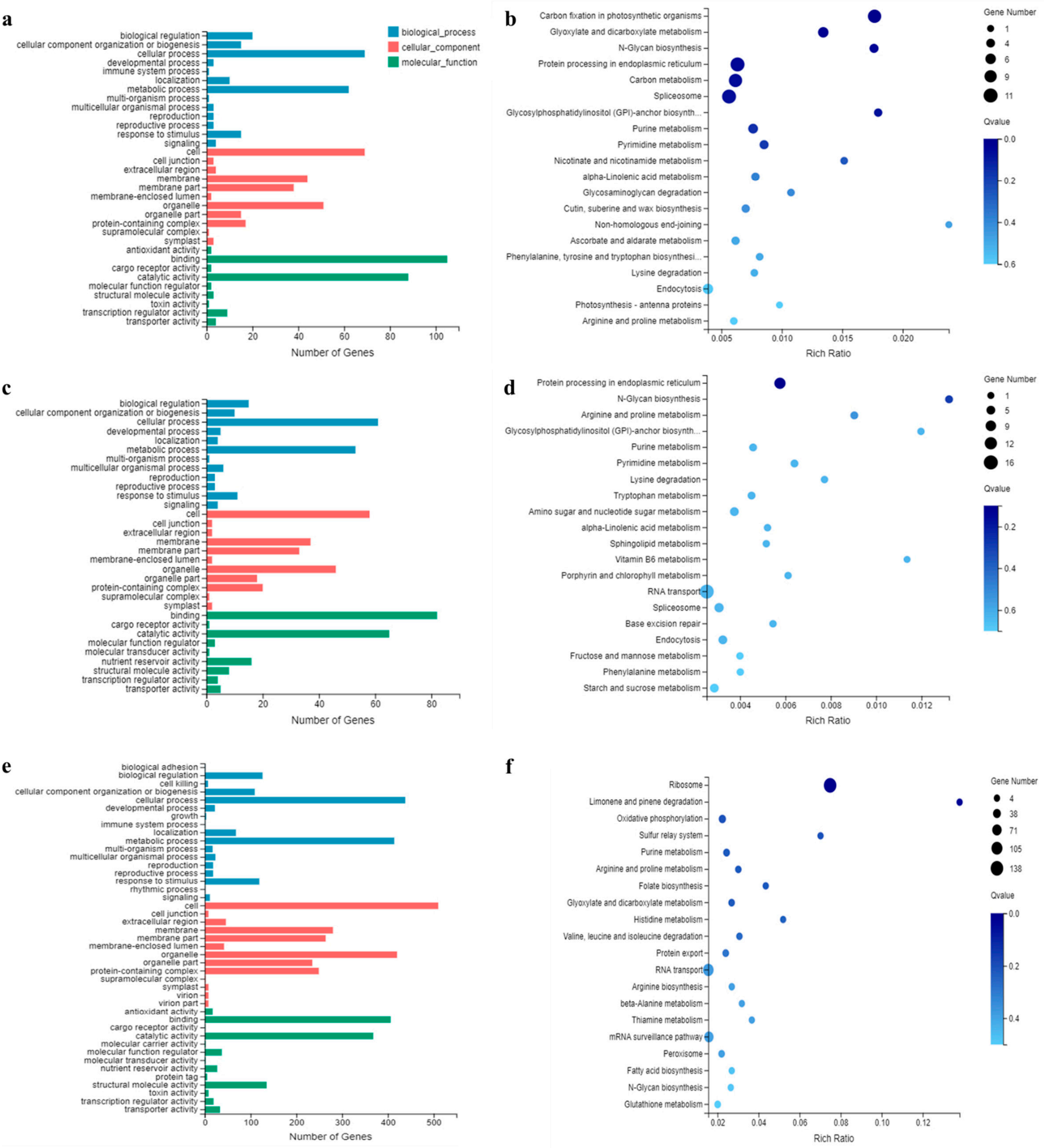

Figure S2 GO and KEGG enrichment of DEGs at different stages. GO enrichment results at 7, 14, and 28 DPA are represented by a, c, and e, respectively. KEGG enrichment results at 7, 14, and 28 DPA are represented by b, d, f, respectively.

a

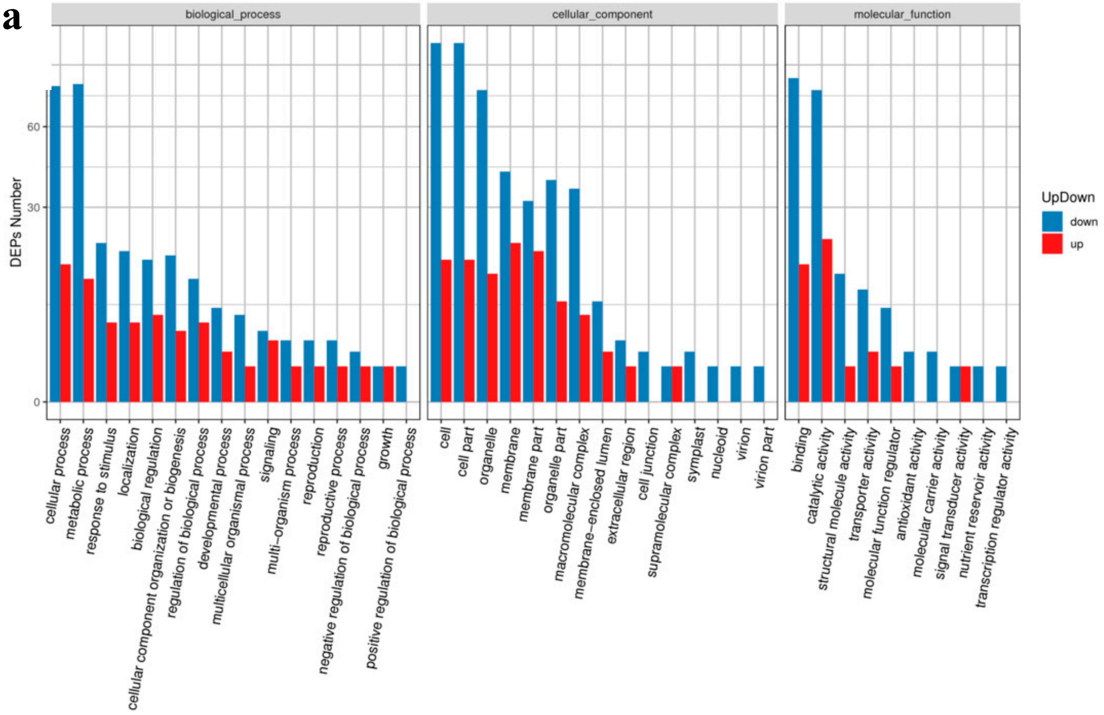
biological_process
cellular_component
molecular_function
DEPs Number
0
30
60
UpDown
down
up
cellular process
metabolic process
response to stimulus
localization
biological regulation
cellular component organization or biogenesis
regulation of biological process
developmental process
multicellular organismal process
signaling
multi−organism process
reproduction
reproductive process
negative regulation of biological process
growth
positive regulation of biological process
cell
cell part
organelle
membrane
membrane part
organelle part
macromolecular complex
membrane−enclosed lumen
extracellular region
cell junction
supramolecular complex
symplast
nucleoid
virion
virion part
binding
catalytic activity
structural molecule activity
transporter activity
molecular function regulator
antioxidant activity
molecular carrier activity
signal transducer activity
nutrient reservoir activity
transcription regulator activity

b

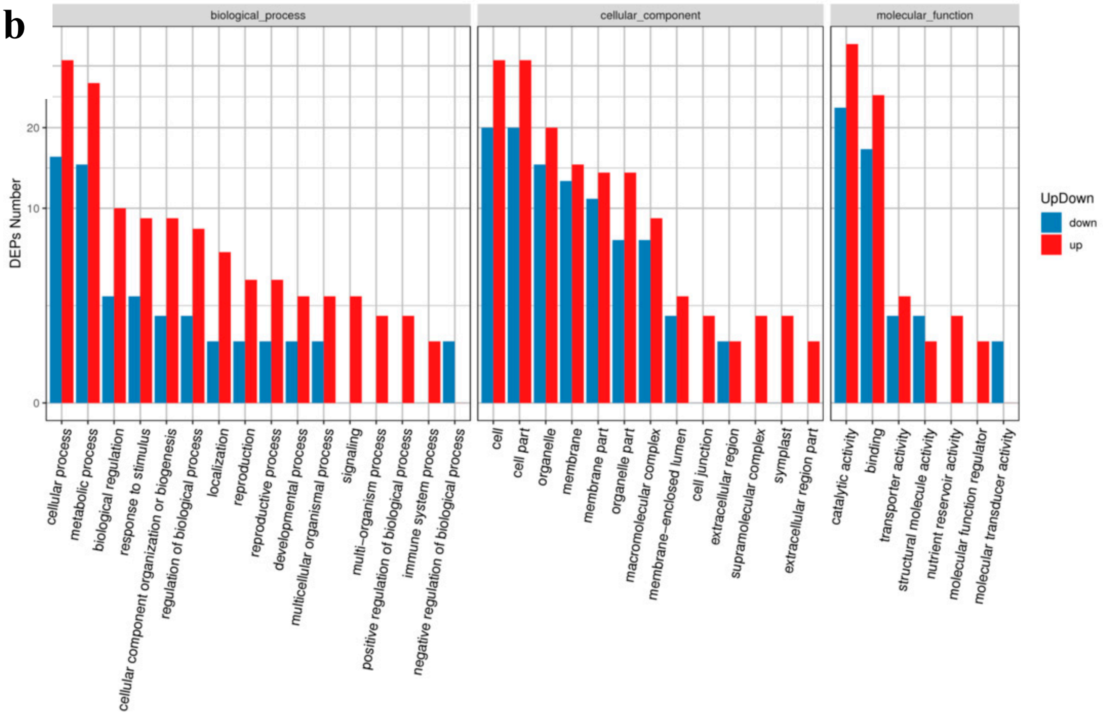
biological_process
cellular_component
molecular_function
DEPs Number
0
10
20
UpDown
down
up
cellular process
metabolic process
biological regulation
response to stimulus
cellular component organization or biogenesis
regulation of biological process
localization
reproduction
reproductive process
developmental process
multicellular organismal process
signaling
multi−organism process
positive regulation of biological process
immune system process
negative regulation of biological process
cell
cell part
organelle
membrane
membrane part
organelle part
macromolecular complex
membrane−enclosed lumen
cell junction
extracellular region
supramolecular complex
symplast
extracellular region part
catalytic activity
binding
transporter activity
structural molecule activity
nutrient reservoir activity
molecular function regulator
molecular transducer activity

c

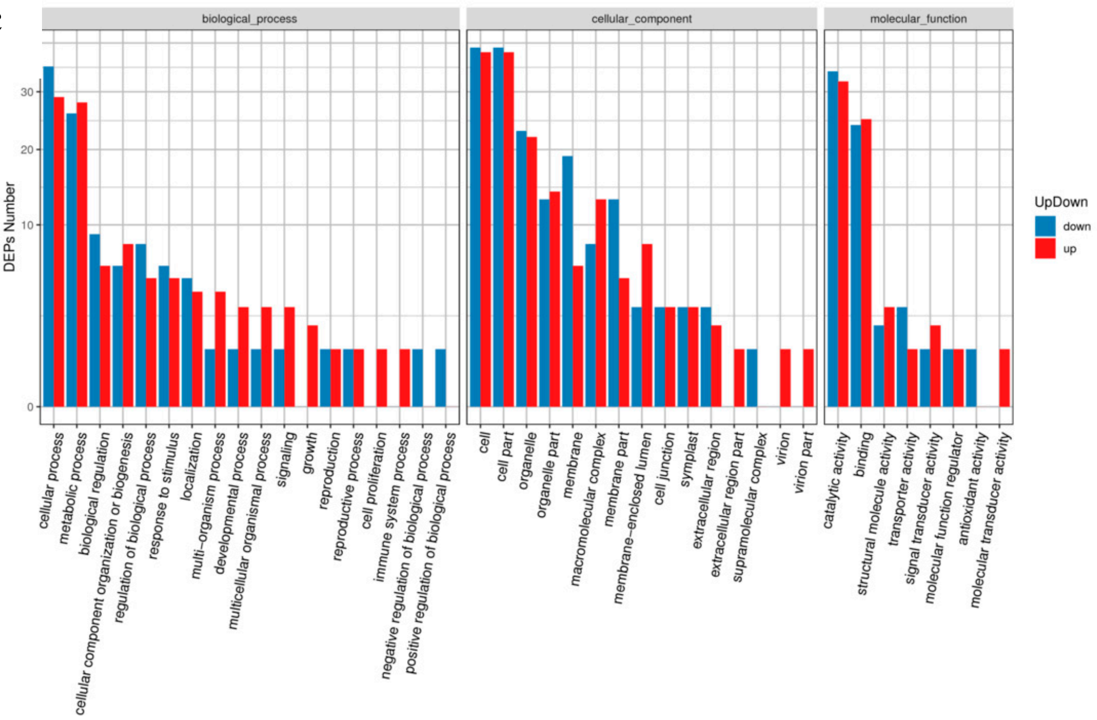

d

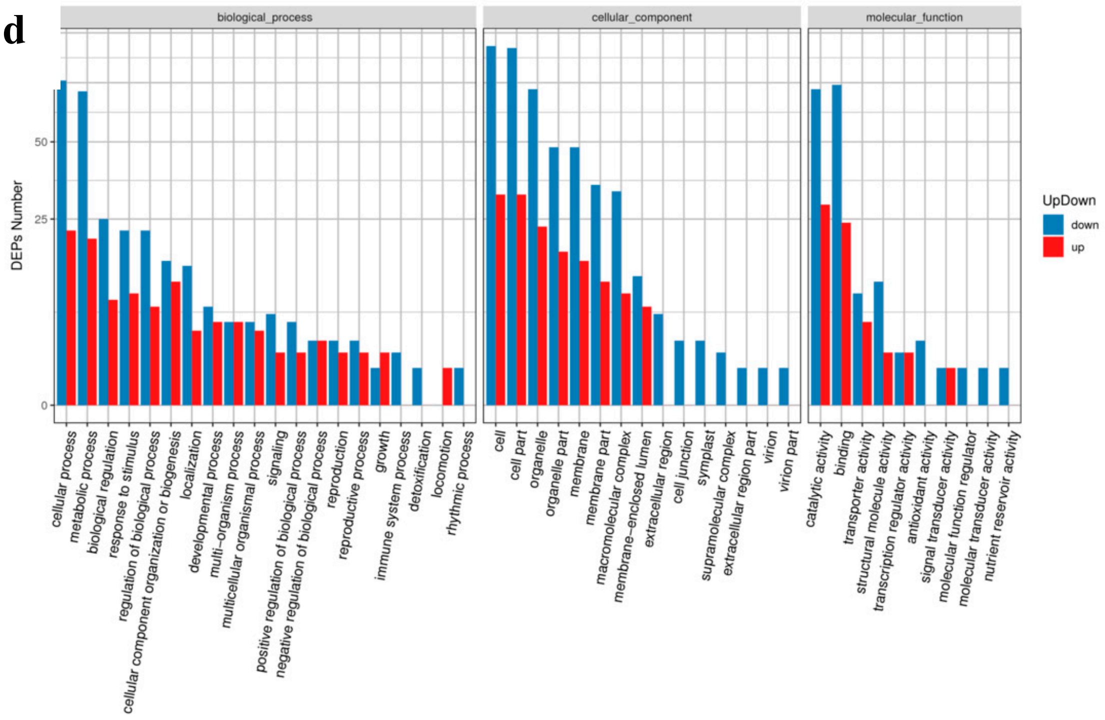

Figure S3 GO and KEGG enrichment of DEPs at different stages. a GO enrichment results of upregulated and downregulated DEPs at 7 DPA; b GO enrichment results of upregulated and downregulated DEPs at 14 DPA; c GO enrichment results of upregulated and downregulated DEPs at 21 DPA; d GO enrichment results of upregulated and downregulated DEPs at 28 DPA.
